# Analysis of body mass index, weight loss and progression of idiopathic pulmonary fibrosis

**DOI:** 10.1186/s12931-020-01528-4

**Published:** 2020-11-25

**Authors:** Stéphane Jouneau, Bruno Crestani, Ronan Thibault, Mathieu Lederlin, Laurent Vernhet, Claudia Valenzuela, Marlies Wijsenbeek, Michael Kreuter, Wibke Stansen, Manuel Quaresma, Vincent Cottin

**Affiliations:** 1 Department of Respiratory Medicine, Competences Centre for Rare Pulmonary Diseases, Pontchaillou Hospital, CHU Rennes, univ Rennes, Rennes 1 University, Rennes, France; 2 Université de Paris, Inserm U1152, APHP, Hôpital Bichat, Centre de reference constitutif pour les maladies pulmonaires rares, Paris, France; 3 INRA, Inserm, Univ Rennes, Nutrition Metabolisms and Cancer, NuMeCan, Unité de Nutrition, CHU Rennes, Rennes, France; 4 Department of Radiology, CHU Rennes, univ Rennes, Rennes, France; 5 Univ Rennes, Inserm, EHESP, Irset (Institut de recherche en santé, environnement et travail) - UMR_S 1085, F-35000 Rennes, France; 6 Pulmonology Department, Hospital Universitario de La Princesa, Madrid, Spain; 7 Department of Respiratory Medicine, Erasmus MC, University Medical Centre, Rotterdam, the Netherlands; 8 Center for Interstitial and Rare Lung Diseases, Pneumology and Respiratory Care Medicine, Thoraxklinik, University of Heidelberg, Member of the German Center for Lung Research, Heidelberg, Germany; 9 Boehringer Ingelheim GmbH & Co. KG, Ingelheim am Rhein, Germany; 10 Boehringer Ingelheim International GmbH, Ingelheim am Rhein, Germany; 11 Reference Center for Rare Pulmonary Diseases, Louis Pradel Hospital, Claude Bernard University Lyon 1, Lyon, France

**Keywords:** Interstitial lung diseases, Treatment, Vital capacity

## Abstract

**Background:**

Nintedanib is an approved therapy for idiopathic pulmonary fibrosis (IPF). Some patients treated with nintedanib experience weight loss. Exploratory data suggest that low body mass index or weight loss are associated with worse outcomes in patients with IPF. We investigated whether BMI at baseline or weight loss over 52 weeks was associated with FVC decline, or influenced the effect of nintedanib, in patients with IPF.

**Methods:**

Using pooled data from the two INPULSIS trials, we analysed the rate of decline in FVC (mL/yr) over 52 weeks in patients treated with nintedanib and placebo in subgroups by baseline BMI (< 25; ≥25 to < 30; ≥30 kg/m^2^) and by weight loss over 52 weeks (≤5; > 5%) using random coefficient regression.

**Results:**

In the placebo group, the mean rate of FVC decline over 52 weeks was numerically greater in patients with lower baseline BMI (− 283.3 [SE 22.4], − 207.9 [20.9] and − 104.5 [21.4] in patients with BMI < 25 kg/m^2^, ≥25 to < 30 kg/m^2^ and ≥ 30 kg/m^2^, respectively). Nintedanib reduced the rate of FVC decline versus placebo in all subgroups by BMI, with a consistent treatment effect across subgroups (interaction *p* = 0.31). In the placebo group, the mean rate of FVC decline was numerically greater in patients with > 5% than ≤5% weight loss over 52 weeks (− 312.7 [SE 32.2] versus − 199.5 [SE 14.4] mL/year). Nintedanib reduced the rate of FVC decline versus placebo in both subgroups by weight loss, with a greater treatment effect in patients with > 5% weight loss (interaction *p* = 0.0008). The adverse event profile of nintedanib was similar across subgroups.

**Conclusions:**

In patients with IPF, lower BMI and weight loss may be associated with faster decline in FVC. Nintedanib reduces the rate of FVC decline both in patients who lose weight on treatment and those who do not.

**Trial registration:**

ClinicalTrials.gov; Nos. NCT01335464 and NCT01335477; URL: www.clinicaltrials.gov.

## Introduction

Idiopathic pulmonary fibrosis (IPF) is an interstitial lung disease characterised by progressive loss of lung function [1]. IPF mainly affects individuals over the age of 60 years and is typically associated with several comorbidities [2]. The clinical course of IPF is variable but ultimately fatal, with a median survival in untreated patients of approximately 3 years from diagnosis [3]. A decline in forced vital capacity (FVC) is an established predictor of mortality in patients with IPF [4].

Some studies have suggested that lower body mass index (BMI) [5–7] or weight loss [8, 9] may be associated with worse prognosis in patients with IPF, although this has not been observed in all studies [10–12]. Large weight loss in obese individuals has been associated with an improvement in FVC [13] and smaller weight reductions with an improvement in FVC in the general population [14]. A retrospective analysis of data from 210 patients with IPF found a greater rate of FVC decline among patients with weight loss > 5% than ≤5% over 1 year [8]. However, it remains unclear whether small weight reductions are associated with changes in FVC in patients with IPF.

Nintedanib is a tyrosine kinase inhibitor approved for the treatment of IPF. In the two Phase III 52-week INPULSIS trials, nintedanib reduced the rate of decline in FVC in patients with mild or moderate impairment in lung function by approximately 50%, with an adverse event profile characterised mainly by gastrointestinal events [15]. Weight loss recorded as an adverse event was more frequently reported in patients treated with nintedanib than placebo [15]. We used pooled data from the INPULSIS trials to investigate whether BMI at baseline or weight loss over 52 weeks was associated with changes in FVC or influenced the treatment effect of nintedanib.

## Methods

### Trial design and participants

The INPULSIS trials (NCT01335464 and NCT01335477) were two randomised, double-blind, placebo-controlled trials of nintedanib in patients with IPF, performed at 205 sites in 24 countries. Eligibility criteria for the INPULSIS trials have been described [15]. Briefly, the participants were aged ≥40 years, with a diagnosis of IPF, FVC ≥50% predicted and diffusing capacity of the lungs for carbon monoxide (DLco) 30–79% predicted.

Patients were randomised 3:2 to receive nintedanib 150 mg twice daily or placebo for 52 weeks, with a follow-up visit 4 weeks after treatment discontinuation. Treatment interruption and dose reduction to 100 mg twice daily were allowed to manage adverse events. After an adverse event had resolved, the dose could be increased back to 150 mg twice daily. Patients who discontinued study drug prematurely were asked to attend all scheduled visits and undergo all examinations as originally planned. FVC was measured at baseline and at weeks 2, 4, 6, 12, 24, 36, and 52 using sponsor-provided spirometers. Weight was measured at baseline and at weeks 2, 4, 6, 12, 24, 36, and 52.

### Outcomes

The pre-specified subgroup analyses of the INPULSIS trials have been reported [15, 16]. Here, we describe post-hoc analyses of efficacy and safety outcomes in subgroups by BMI at baseline and by weight loss over 52 weeks. Efficacy outcomes assessed were the annual rate of decline in FVC (mL/year); absolute change from baseline in FVC (mL); absolute change from baseline in FVC % predicted; time to first investigator-reported acute exacerbation; absolute change from baseline in St. George’s Respiratory Questionnaire (SGRQ) total score; time to absolute decline in FVC ≥10% predicted or death; and time to death, all over 52 weeks. An acute exacerbation was defined based on worsening or development of dyspnoea and the appearance of new abnormalities on high-resolution computed tomography (HRCT), with the exclusion of known causes of acute worsening in respiratory function [15]. The SGRQ is a self-administered questionnaire, comprising three domains (symptoms, activity, impact), which assesses health-related quality of life in patients with respiratory disease on a scale of 0 to 100 [17]. Safety was assessed based on adverse events reported by the investigators (irrespective of causality) with onset after the first dose and up to 4 weeks after the last dose of study drug, which were coded according to preferred terms in the Medical Dictionary for Regulatory Activities (MedDRA) version 20.1. Reductions in weight reported by the investigators as adverse events (based on perceived clinical relevance rather than a defined degree of weight loss) were coded under the preferred term “weight decreased”.

### Statistical analysis

Analyses were conducted using data from patients who received ≥1 dose of nintedanib or placebo. In the primary analysis, the annual rate of decline in FVC (mL/year) was analysed using a random coefficient regression model (with random slopes and intercepts) with fixed effects for trial, treatment, sex, age and height and random effect of patient-specific intercept and time. To assess the effect of BMI at baseline or weight loss on the treatment effect of nintedanib, we repeated the original analyses with the addition of covariates for race (White; Asian; Black/African-American) and either BMI at baseline (< 25; ≥25 to < 30; ≥30 kg/m^2^) or weight loss over 52 weeks (weight gain or ≤ 5% weight loss; > 5% weight loss) to the model. In addition, we assessed outcomes in subgroups by BMI at baseline (< 25 kg/m^2^; ≥25 to < 30 kg/m^2^; ≥30 kg/m^2^) and by weight loss over 52 weeks (weight gain or ≤ 5% weight loss; > 5% weight loss). Weight loss ≤5% or > 5% over 52 weeks was assessed based on the annual rate of decline in weight analysed using a random coefficient regression model with fixed effects for trial, treatment, sex, age and height and random effect of patient specific-intercept and time. We also analysed the annual rate of decline in FVC (mL/year) in subgroups based on BMI below and at least the median at baseline, and in subgroups by weight loss over 52 weeks (weight gain/no weight loss; > 0 to ≤5% weight loss; > 5 to ≤10% weight loss; > 10% weight loss) based on the change from baseline in weight at week 52 (or, if no week 52 measurement was available, the last measurement after baseline but before week 52). In analyses of the annual rate of decline in FVC, the term subgroup and the interaction terms treatment-by-subgroup, time-by-subgroup and treatment-by-time-by-subgroup were included in the model. The model allowed for missing data, assuming that they were missing at random; missing data were not imputed. The interaction *p*-values are an indicator of the difference in the effect of nintedanib versus placebo among the subgroups. The other statistical analyses are described in Supplemental Appendix 1. Analyses were not adjusted for multiplicity. Adverse events are presented descriptively.

## Results

### Patients

A total of 638 patients with IPF were treated with nintedanib and 423 with placebo. Baseline characteristics were balanced between the nintedanib and placebo groups. Most (79.3%) of the participants were male. Mean (SD) age at baseline was 66.8 (8.0) years, weight was 79.0 (16.6) kg, BMI was 27.9 (4.6) kg/m^2^, and FVC % predicted was 79.6 (17.8).

### Analyses based on BMI at baseline

At baseline, 307 (28.9%), 453 (42.7%) and 301 (28.4%) patients had BMI < 25 kg/m^2^, ≥25 to < 30 kg/m^2^ and ≥ 30 kg/m^2^, respectively. Compared with patients with BMI ≥30 kg/m^2^, greater proportions of patients with BMI < 25 or ≥ 25 to < 30 kg/m^2^ were of Asian race, and greater proportions with BMI < 25 kg/m^2^ had never smoked (Supplemental Table 1). Low correlation was observed between BMI at baseline and FVC (mL) at baseline (Supplemental Figure 1).

**Fig. 1:**
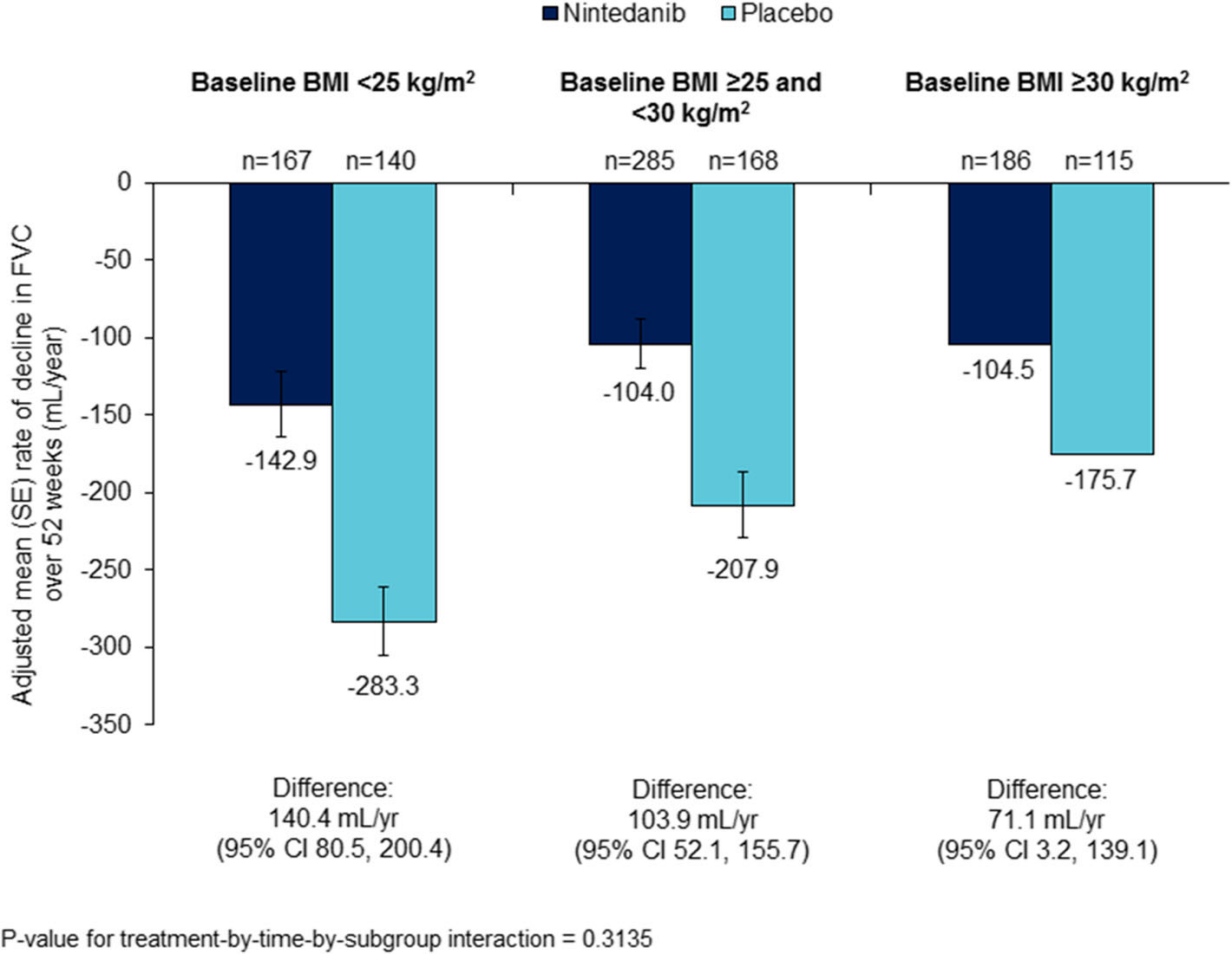
Annual rate of decline in FVC (mL/year) assessed over 52 weeks by BMI at baseline

**Fig. 2:**
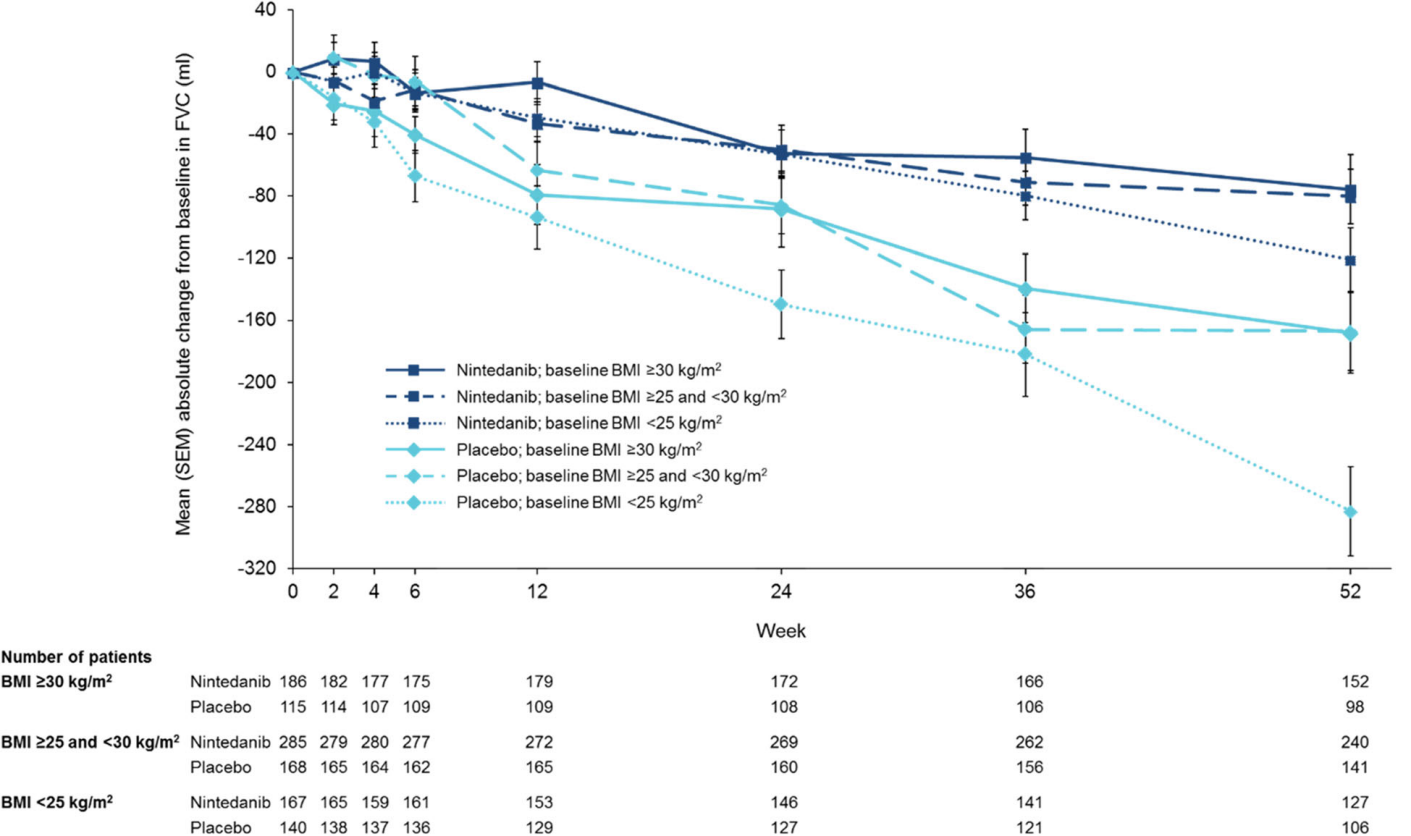
Change from baseline in FVC (mL) over 52 weeks by BMI at baseline

Low correlation was observed between BMI at baseline and the rate of decline in FVC over 52 weeks (Supplemental Figure 1). However, in the placebo group, the mean rate of decline in FVC (mL/year) over 52 weeks was numerically greater in patients with BMI < 25 kg/m^2^ (− 283.3 [SE 22.4]) than BMI ≥25 to < 30 kg/m^2^ (− 207.9 [20.9]) or BMI ≥30 kg/m^2^ (− 175.7 [27.0]) (Figs. 1 and 2; Table 1). In the nintedanib group, the mean rate of decline in FVC over 52 weeks was similar across subgroups with baseline BMI < 25 kg/m^2^, ≥25 to < 30 kg/m^2^ and ≥ 30 kg/m^2^ (mL/year) (− 142.9 [SE 20.8], − 104.0 [16.0] and − 104.5 [21.4] mL/year, respectively) (Figs. 1 and 2; Table 1). Nintedanib reduced the annual rate of decline in FVC versus placebo in all the subgroups by baseline BMI with a treatment effect that was numerically greater in patients with lower BMI, but the *p*-value for treatment-by-time-by-subgroup interaction did not indicate heterogenous treatment effects across the subgroups (*p* = 0.31). Similar results were observed when the rate of decline in FVC was analysed in subgroups by baseline BMI above and below the median (27 kg/m^2^) (Supplemental Table 2). When BMI at baseline (< 25; ≥25 to < 30; ≥30 kg/m^2^) and race were included in the model analysing the rate of decline in FVC (mL/year) over 52 weeks, the difference between treatment groups was consistent with the original analysis (Supplemental Table 3).

**Table 1:** Outcomes in subgroups of patients by BMI at baseline

	BMI < 25 kg/m^**2**^ at baseline		BMI ≥ 25 and < 30 kg/m^**2**^ at baseline		BMI ≥ 30 kg/m^**2**^ at baseline	
	Nintedanib (***n*** = 167)	Placebo (***n*** = 140)	Nintedanib (***n*** = 285)	Placebo (***n*** = 168)	Nintedanib (***n*** = 186)	Placebo (***n*** = 115)
Annual rate of decline in FVC (mL/year) over 52 weeks	−142.9 (20.8)	−283.3 (22.4)	−104.0 (16.0)	−207.9 (20.9)	− 104.5 (21.4)	−175.7 (27.0)
Difference versus placebo (95% CI)	140.4 (80.5, 200.4)		103.9 (52.1, 155.7)		71.1 (3.2, 139.1)	
*p*-value for treatment-by-time-by-subgroup interaction	0.31					
Absolute change from baseline in FVC (mL) over 52 weeks	−142.1 (23.1)	− 295.4 (25.1)	− 94.1 (17.6)	−175.4 (22.9)	−95.0 (21.5)	− 175.7 (27.1)
Difference versus placebo (95% CI)	153.4 (86.2, 220.5)		81.2 (24.4, 138.0)		80.8 (12.5, 149.0)	
*p*-value for treatment-by-subgroup interaction	0.16					
Absolute change from baseline in FVC (% predicted) over 52 weeks	−4.7 (0.7)	− 9.0 (0.8)	−2.8 (0.5)	− 5.1 (0.7)	−2.6 (0.6)	− 5.1 (0.7)
Difference versus placebo (95% CI)	4.3 (2.2, 6.4)		2.2 (0.6, 3.9)		2.6 (0.7, 4.4)	
*p*-value for treatment-by-subgroup interaction	0.11					
Absolute change from baseline in SGRQ total score over 52 weeks	5.2 (1.3)	7.0 (1.4)	3.4 (1.0)	4.5 (1.3)	2.9 (1.2)	4.2 (1.5)
Difference versus placebo (95% CI)	−1.8 (− 5.6, 2.0)		−1.1 (−4.4, 2.2)		− 1.3 (− 5.0, 2.3)	
*p*-value for treatment-by-subgroup interaction	0.45					
Patients with absolute decline in FVC ≥10% predicted or death at week 52, n (%)	52 (31.1)	69 (49.3)	70 (24.6)	60 (35.7)	51 (27.4)	46 (40.0)
HR (95% CI)	0.48 (0.33, 0.69)		0.68 (0.48, 0.96)		0.62 (0.41, 0.92)	
*p*-value for treatment-by-subgroup interaction	0.58					
Patients with ≥1 acute exacerbation of IPF over 52 weeks	11 (6.6)	11 (7.9)	15 (5.3)	13 (7.7)	5 (2.7)	8 (7.0)
HR (95% CI)	0.81 (0.35, 1.91)		0.69 (0.32, 1.45)		0.42 (0.14, 1.30)	
*p*-value for treatment-by-subgroup interaction	0.64					
Deaths over 52 weeks, n (%)	10 (6.0)	15 (10.7)	16 (5.6)	10 (6.0)	9 (4.8)	8 (7.0)
HR (95% CI)	0.49 (0.22, 1.11)		0.92 (0.42, 2.04)		0.69 (0.27, 1.81)	
*p*-value for treatment-by-subgroup interaction	0.60					

Changes from baseline are adjusted mean (SE). *HR* Hazard ratio

The mean increase (worsening) in SGRQ total score at week 52 in placebo-treated patients was numerically greater in patients with baseline BMI < 25 kg/m^2^ than ≥25 to < 30 kg/m^2^ and ≥ 30 kg/m^2^ (7.0 versus 4.5 and 4.2, respectively). The proportions of placebo-treated patients who had an investigator-reported acute exacerbation was similar across these subgroups (7.9, 7.7 and 7.0%, respectively), while the proportions of patients who died was numerically greater in patients with baseline BMI < 25 kg/m^2^ than ≥25 to < 30 kg/m^2^ or ≥ 30 kg/m^2^ (10.7% versus 6.0 and 7.0%, respectively).

Differences between the nintedanib and placebo groups in absolute changes from baseline in FVC (mL and % predicted) and change in SGRQ total score were numerically greater in patients with BMI < 25 kg/m^2^ than ≥25 to < 30 kg/m^2^ or ≥ 30 kg/m^2^, but the *p*-values for treatment-by-subgroup interaction did not indicate heterogenous treatment effects across the subgroups (Table 1). Differences between the nintedanib and placebo groups in the proportion of patients with an absolute decline in FVC ≥10% predicted or death, and the proportion of patients who died were consistent across subgroups by BMI at baseline (Table 1).

### Analyses based on weight loss over 52 weeks

The mean (SE) annual rate of decline in weight over 52 weeks was − 3.3 (0.2) kg/year and − 1.5 (0.2) kg/year in the nintedanib and placebo groups, respectively. Based on the annual rate of decline in weight, in the nintedanib group, 397 (62.2%) patients had weight gain or ≤ 5% weight loss and 241 (37.8%) had > 5% weight loss over 52 weeks, and in the placebo group, 338 (79.9%) had weight gain or ≤ 5% weight loss and 85 (20.1%) had > 5% weight loss over 52 weeks. Compared with patients with ≤5% weight loss, patients with > 5% weight loss over 52 weeks had a greater proportion of females, lower mean FVC and DLco % predicted at baseline, and higher (worse) mean SGRQ total score at baseline (Supplemental Table 4).

**Fig. 3:**
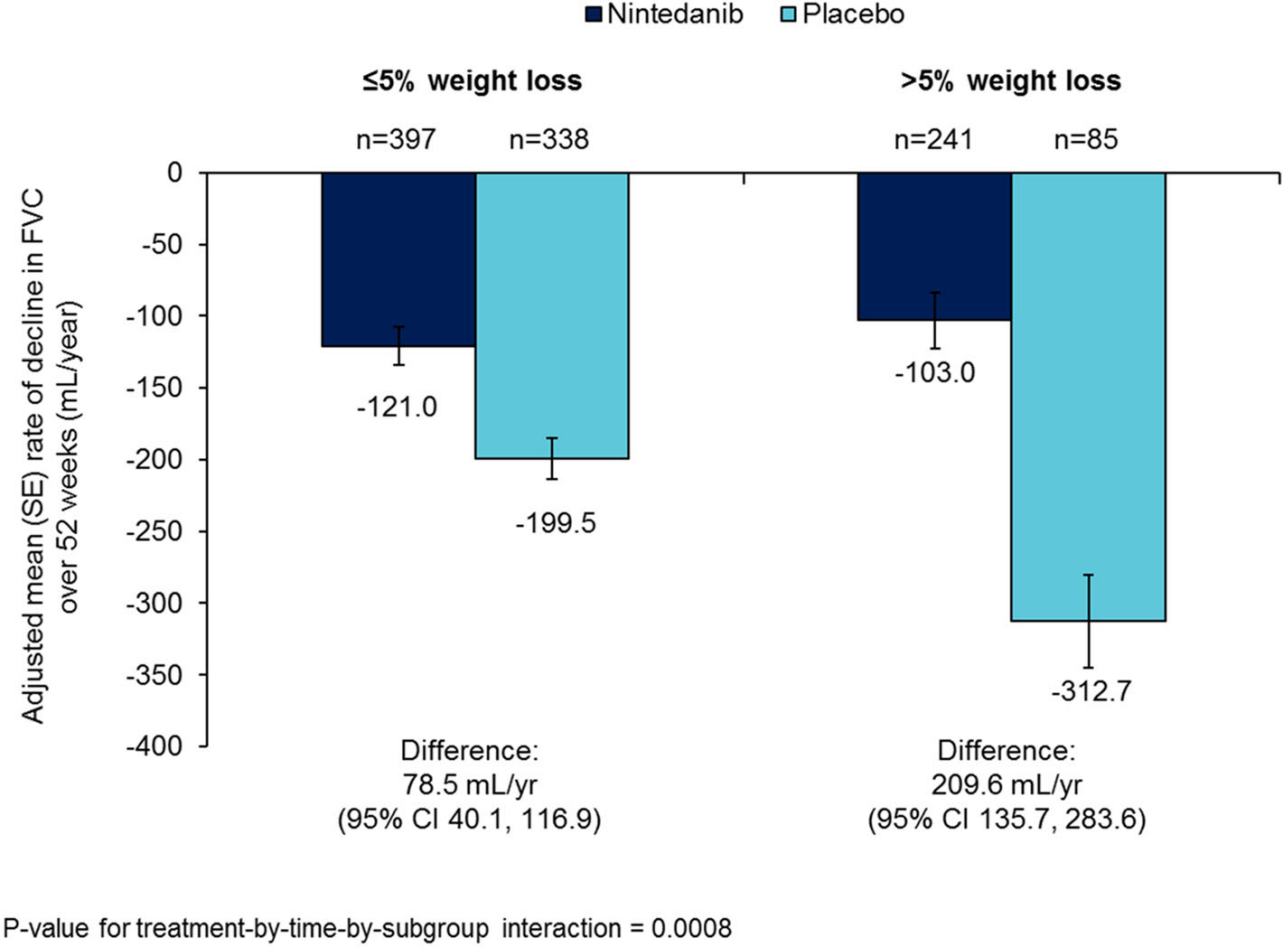
Annual rate of decline in FVC (mL/year) over 52 weeks by weight loss over 52 weeks

**Fig. 4:**
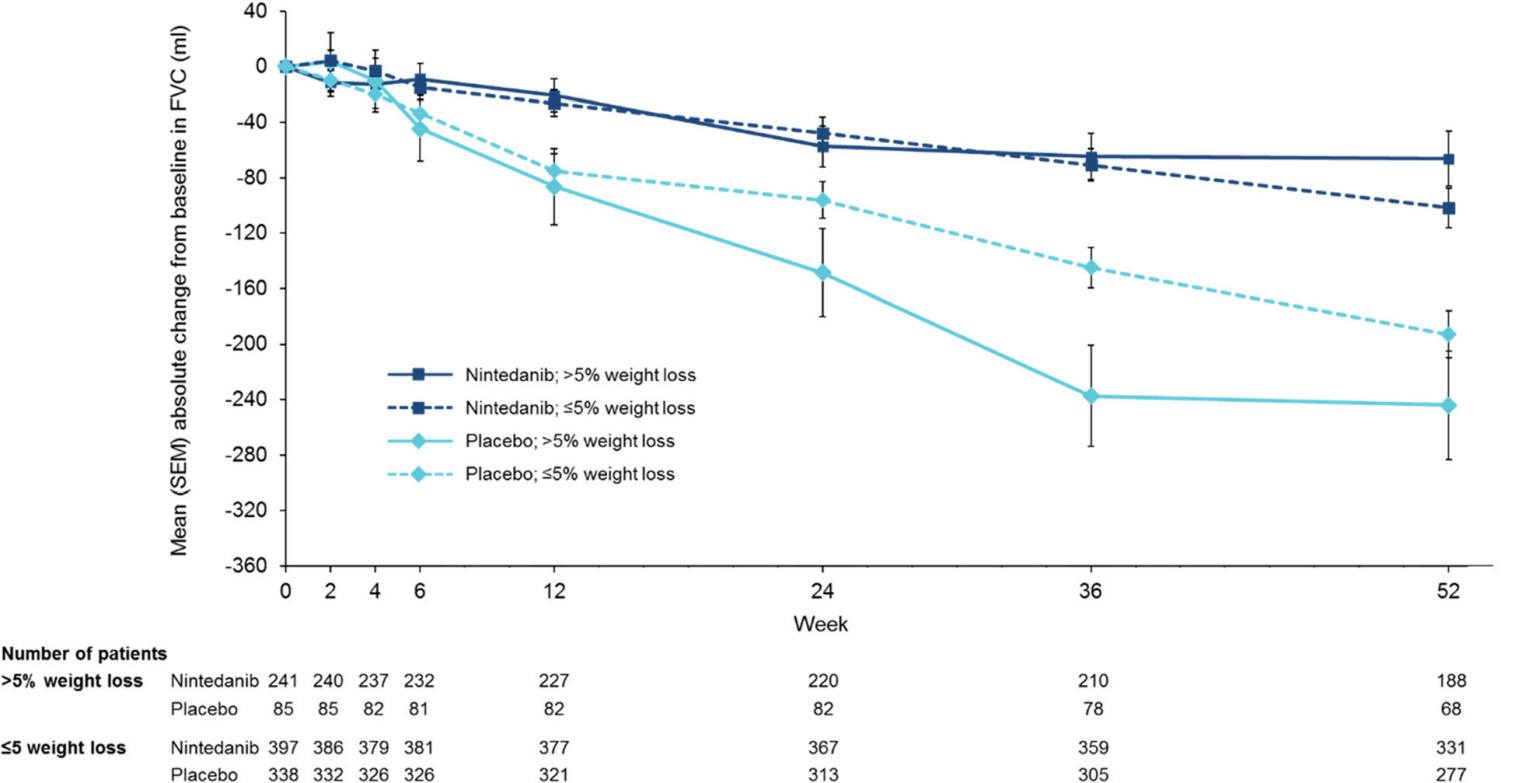
Change from baseline in FVC (mL) over 52 weeks by weight loss over 52 weeks

In the placebo group, the rate of decline in FVC (mL/year) was numerically greater in patients with > 5% than ≤5% weight loss over 52 weeks (− 312.7 [SE 32.2] versus − 199.5 [SE 14.4] mL/year) (Figs. 3 and 4; Table 2), as was the mean increase (worsening) in SGRQ total score at week 52 (13.6 versus 3.0). In the placebo group, the proportion of patients who had an acute exacerbation was numerically greater (11.8% versus 6.5%), but the proportion of patients who died was numerically lower (5.9% versus 8.3%), in patients with > 5% than ≤5% weight loss.

**Table 2:** Outcomes in subgroups of patients by weight loss over 52 weeks^a^

	Weight loss ≤ 5%		Weight loss > 5%	
	Nintedanib (***n*** = 397)	Placebo (***n*** = 338)	Nintedanib (***n*** = 241)	Placebo (***n*** = 85)
Annual rate of decline in FVC (mL/year) assessed over 52 weeks	− 121.0 (13.2)	−199.5 (14.4)	− 103.0 (19.5)	−312.7 (32.2)
Difference versus placebo (95% CI)	78.5 (40.1, 116.9)		209.6 (135.7, 283.6)	
*p*-value for treatment-by-time-by-subgroup interaction	0.0008			
Absolute change from baseline in FVC (mL) over 52 weeks	− 114.2 (14.6)	− 197.3 (15.9)	−94.4 (20.1)	− 278.9 (33.4)
Difference versus placebo (95% CI)	83.1 (40.8, 125.4)		184.5 (107.8, 261.2)	
*p*-value for treatment-by-subgroup interaction	0.54			
Absolute change from baseline in FVC % predicted over 52 weeks	−3.3 (0.4)	−5.6 (0.5)	−3.1 (0.6)	−9.1 (1.0)
Difference versus placebo (95% CI)	2.3 (1.1, 3.5)		6.0 (3.6, 8.4)	
*p-*value for treatment-by-subgroup interaction	0.54			
Absolute change from baseline in SGRQ total score over 52 weeks	3.3 (0.8)	3.0 (0.9)	4.2 (1.1)	13.6 (1.7)
Difference versus placebo (95% CI)	0.3 (−2.1, 2.7)		−9.5 (−13.5, −5.4)	
*p*-value for treatment-by-subgroup interaction	0.20			
Patients with absolute decline in FVC ≥10% predicted or death at week 52, n (%)	100 (25.2)	128 (37.9)	73 (30.3)	47 (55.3)
HR (95% CI)	0.62 (0.48, 0.81)		0.51 (0.35, 0.73)	
*p*-value for treatment-by-subgroup interaction	0.32			
Patients with ≥1 acute exacerbation of IPF over 52 weeks	14 (3.5)	22 (6.5)	17 (7.1)	10 (11.8)
HR (95% CI)	0.53 (0.27, 1.04)		0.63 (0.29, 1.38)	
*p*-value for treatment-by-subgroup interaction	0.84			
Deaths over 52 weeks, n (%)	19 (4.8)	28 (8.3)	16 (6.6)	5 (5.9)
HR (95% CI)	0.58 (0.32, 1.04)		1.24 (0.45, 3.41)	
*p*-value for treatment-by-subgroup interaction	0.23			

Changes from baseline are adjusted mean (SE). *HR* Hazard ratio. ^a^Based on the annual rate of decline in weight

When weight loss over 52 weeks and race were included in the model analysing the annual rate of decline in FVC (mL/year), the difference between nintedanib and placebo was consistent with the original analysis (Supplemental Table 3). Nintedanib reduced the annual rate of decline in FVC versus placebo both in patients with ≤5 and > 5% weight loss over 52 weeks, with a greater effect in patients with > 5% than ≤5% weight loss (*p* = 0.0008 for treatment-by-time-by-subgroup interaction) (Figs. 3 and 4; Table 2). Nintedanib also had a greater effect on the annual rate of decline in FVC in patients with greater weight loss based on the change from baseline in weight at week 52 (no weight loss, > 0 to ≤5% weight loss, > 5 to ≤10% weight loss and > 10% weight loss) (*p* = 0.0017 for treatment-by-time-by-subgroup interaction) (Supplemental Figure 2). Differences between the nintedanib and placebo groups in change in SGRQ total score and the proportion of patients with an absolute decline in FVC ≥10% predicted or death were numerically greater in patients with > 5% than ≤5% weight loss, but the *p*-values for treatment-by-subgroup interaction did not indicate heterogenous treatment effects between the subgroups (Table 2).

### Adverse events in subgroups by BMI at baseline and weight loss over 52 weeks

In both the nintedanib and placebo groups, the proportions of patients with adverse events of decreased appetite, weight decrease, and progression of IPF were greater in patients with lower baseline BMI and in patients with > 5% than ≤5% weight loss over 52 weeks (Tables 3 and 4). The proportion of patients with diarrhoea adverse events was greater in those with > 5% than ≤5% weight loss (Table 4). The adverse event profile of nintedanib was similar across subgroups by baseline BMI and weight loss over 52 weeks, with gastrointestinal adverse events reported more frequently in patients treated with nintedanib than placebo (Tables 3 and 4).

**Table 3:** Adverse events (reported irrespective of causality) in subgroups of patients by BMI at baseline

	BMI < 25 kg/m^**2**^ at baseline		BMI ≥ 25 and < 30 kg/m^**2**^ at baseline		BMI ≥ 30 kg/m^**2**^ at baseline	
	Nintedanib (***n*** = 167)	Placebo (***n*** = 140)	Nintedanib (***n*** = 285)	Placebo (***n*** = 168)	Nintedanib (***n*** = 186)	Placebo (***n*** = 115)
Adverse event(s)	161 (96.4)	124 (88.6)	270 (94.7)	151 (89.9)	178 (95.7)	104 (90.4)
Most frequent adverse event(s)^a^						
Diarrhoea	102 (61.1)	24 (17.1)	176 (61.8)	33 (19.6)	115 (61.8)	21 (18.3)
Nausea	37 (22.2)	7 (5.0)	71 (24.9)	11 (6.5)	48 (25.8)	10 (8.7)
Progression of IPF^b^	25 (15.0)	28 (20.0)	28 (9.8)	25 (14.9)	11 (5.9)	8 (7.0)
Nasopharyngitis	33 (19.8)	21 (15.0)	32 (11.2)	32 (19.0)	22 (11.8)	15 (13.0)
Cough	16 (9.6)	16 (11.4)	38 (13.3)	32 (19.0)	31 (16.7)	9 (7.8)
Decreased appetite	26 (15.6)	13 (9.3)	23 (8.1)	11 (6.5)	19 (10.2)	0
Vomiting	25 (15.0)	7 (5.0)	25 (8.8)	1 (0.6)	24 (12.9)	3 (2.6)
Bronchitis	12 (7.2)	8 (5.7)	32 (11.2)	20 (11.9)	23 (12.4)	17 (14.8)
Dyspnoea	9 (5.4)	14 (10.0)	23 (8.1)	19 (11.3)	17 (9.1)	15 (13.0)
Weight decreased	19 (11.4)	5 (3.6)	28 (9.8)	8 (4.8)	15 (8.1)	2 (1.7)
Upper respiratory tract infection	17 (10.2)	14 (10.0)	23 (8.1)	17 (10.1)	18 (9.7)	11 (9.6)
Fatigue	9 (5.4)	14 (10.0)	14 (4.9)	13 (7.7)	17 (9.1)	6 (5.2)
Adverse event(s) leading to treatment discontinuation	43 (25.7)	19 (13.6)	50 (17.5)	19 (11.3)	30 (16.1)	17 (14.8)
Severe adverse event(s)^c^	44 (26.3)	34 (24.3)	81 (28.4)	42 (25.0)	49 (26.3)	23 (20.0)
Serious adverse event(s)^d^	61 (36.5)	37 (26.4)	82 (28.8)	54 (32.1)	51 (27.4)	36 (31.3)
Fatal adverse event(s)	8 (4.8)	13 (9.3)	18 (6.3)	9 (5.4)	11 (5.9)	9 (7.8)

Data are n (%) of patients with ≥1 such adverse event reported over 52 weeks plus a 4-week post-treatment follow-up period. ^a^Adverse events by MedDRA preferred term reported in ≥10% of patients in ≥1 of the subgroups shown. ^b^Corresponds to MedDRA term ‘IPF’, which included disease worsening and acute exacerbations. ^c^Event that was incapacitating or that caused an inability to work or to perform usual activities. ^d^Event that resulted in death, was immediately life-threatening, resulted in persistent or clinically significant disability or incapacity, required or prolonged hospitalisation, was related to a congenital anomaly or birth defect, or was deemed serious for any other reason

**Table 4:** Adverse events (reported irrespective of causality) in subgroups of patients by weight loss over 52 weeks^a^

	Weight loss ≤ 5%		Weight loss > 5%	
	Nintedanib (***n*** = 397)	Placebo (***n*** = 338)	Nintedanib (***n*** = 241)	Placebo (***n*** = 85)
Adverse event(s)	376 (94.7)	297 (87.9)	233 (96.7)	82 (96.5)
Most frequent adverse event(s)^b^				
Diarrhoea	216 (54.4)	56 (16.6)	177 (73.4)	22 (25.9)
Progression of IPF^c^	30 (7.6)	41 (12.1)	34 (14.1)	20 (23.5)
Dyspnoea	24 (6.0)	37 (10.9)	25 (10.4)	11 (12.9)
Nausea	90 (22.7)	20 (5.9)	66 (27.4)	8 (9.4)
Pneumonia	21 (5.3)	14 (4.1)	12 (5.0)	12 (14.1)
Nasopharyngitis	46 (11.6)	59 (17.5)	41 (17.0)	9 (10.6)
Decreased appetite	31 (7.8)	14 (4.1)	37 (15.4)	10 (11.8)
Cough	57 (14.4)	45 (13.3)	28 (11.6)	12 (14.1)
Bronchitis	43 (10.8)	33 (9.8)	24 (10.0)	12 (14.1)
Vomiting	39 (9.8)	9 (2.7)	35 (14.5)	2 (2.4)
Upper respiratory tract infection	40 (10.1)	33 (9.8)	18 (7.5)	9 (10.6)
Abdominal pain	31 (7.8)	8 (2.4)	25 (10.4)	2 (2.4)
Weight decreased	14 (3.5)	3 (0.9)	48 (19.9)	12 (14.1)
Arthralgia	8 (2.0)	12 (3.6)	6 (2.5)	9 (10.6)
Adverse event(s) leading to treatment discontinuation	72 (18.1)	38 (11.2)	51 (21.2)	17 (20.0)
Severe adverse event(s)^d^	94 (23.7)	72 (21.3)	80 (33.2)	27 (31.8)
Serious adverse event(s)^e^	97 (24.4)	86 (25.4)	97 (40.2)	41 (48.2)
Fatal adverse event(s)	20 (5.0)	25 (7.4)	17 (7.1)	6 (7.1)

Data are n (%) of patients with ≥1 such adverse event reported over 52 weeks plus a 4-week post-treatment follow-up period. ^a^Based on the annual rate of decline in weight. ^b^Adverse events by MedDRA preferred term reported in ≥10% of patients in ≥1 of the subgroups shown. ^c^Corresponds to MedDRA term ‘IPF’, which included disease worsening and acute exacerbations. ^d^Event that was incapacitating or that caused an inability to work or to perform usual activities. ^e^Event that resulted in death, was immediately life-threatening, resulted in persistent or clinically significant disability or incapacity, required or prolonged hospitalisation, was related to a congenital anomaly or birth defect, or was deemed serious for any other reason

## Discussion

These *post-hoc* analyses of pooled data from the INPULSIS trials suggest that the rate of decline in FVC over 52 weeks was greater in untreated patients with IPF who had lower BMI at baseline, and in patients who had > 5% weight loss during the trials. The effect of nintedanib on reducing the rate of FVC decline was numerically more pronounced in patients with lower BMI at baseline and in patients who had > 5% weight loss during the trials. The rate of decline in FVC in nintedanib-treated patients was similar between the subgroups by baseline BMI and by weight loss during the trials.

Our finding that weight loss was associated with a faster decline in FVC is consistent with a retrospective analysis of two cohorts of patients with IPF (a Japanese cohort of 124 patients and a UK cohort of 86 patients), which found a greater rate of FVC decline in patients with weight loss > 5% than ≤5% over 1 year [8]. In our analyses, weight loss > 5% over 52 weeks was also associated with a greater worsening in health-related quality of life assessed using the SGRQ. The reasons for this are not clear, but it may be that this extent of weight loss reflects a significant worsening in a patient’s overall health (not necessarily related to their IPF). A weight loss of 5% is often used to define significant unintentional weight loss and is included in the definition of cachexia [18, 19] and in criteria for the diagnosis of malnutrition proposed by a global consensus group [20].

Low BMI and low fat-free mass are common in patients with IPF [21]. Weight loss in patients with IPF may occur due to physical inactivity [22], which leads to loss of muscle mass, or to loss of appetite due to factors such as symptoms, medications, malnutrition, or depression [23]. In our analyses, greater proportions of patients who had > 5% than ≤5% weight loss during the trials had adverse events of diarrhoea and decreased appetite (in both the nintedanib and placebo groups). Antifibrotic therapies (nintedanib and pirfenidone) are associated with gastrointestinal adverse events, including diarrhoea, nausea, and vomiting, and with loss of appetite and weight loss [24, 25]. Almost 20% of patients in the placebo group and over a third of patients in the nintedanib group had a weight loss > 5% over 52 weeks, highlighting the importance of supportive care, including nutritional interventions when required and measures to avoid physical inactivity and loss of muscle mass, in patients with this debilitating disease. In most patients, gastrointestinal effects of antifibrotic therapy can be managed successfully with hydration and symptomatic therapy or, if this is insufficient, by dose adjustment [26–28].

Modelling of pharmacokinetic data from patients with IPF treated with nintedanib showed that age, weight, Asian race, smoking, and lactate dehydrogenase levels have small to moderate effects on nintedanib exposure, which are within the range of inter-patient variability and do not warrant a priori dose adjustment [29, 30]. As such, it is unlikely that the numerically greater effect of nintedanib on FVC decline observed in patients with lower BMI is explained by the higher nintedanib exposure expected in this subgroup, although a contribution of pharmacokinetics to this finding cannot be ruled out. Previous subgroup analyses of data from the INPULSIS trials have shown that despite Asian patients having a lower BMI and lower FVC, there was no difference in the effect of nintedanib on FVC decline, or the adverse event profile of nintedanib, between Asian and White patients [31, 32].

Strengths of our analyses include the large sample size (1061 patients with a diagnosis of IPF confirmed by the investigator) and the relatively small amount of missing data on FVC, BMI and weight loss. Our analyses also have several limitations, including that they were *post-hoc*, that the duration of follow-up was only 52 weeks, that the reasons for weight loss could not be determined, that there were differences between the subgroups by BMI and weight loss beyond factors that could be adjusted for, and that there were too few underweight (BMI < 18.5 kg/m^2^) patients (*n* = 8) for them to be analysed as a subgroup. While our analyses indicate potential associations between BMI or weight loss and FVC decline, and it is possible that there are links between weight loss and fibrotic processes, we have not demonstrated causal relationships. The limitations of BMI and weight loss as measures of nutritional status and general health must be considered. Patients’ weight loss prior to enrolment in the INPULSIS trials is unknown.

## Conclusions

These analyses of data from the INPULSIS trials showed that patients with IPF who received placebo and had a lower BMI at baseline, or a weight loss > 5% over 52 weeks, had a greater annual rate of decline in FVC. Nintedanib reduced the rate of decline in FVC both in patients with lower and higher BMI, and in patients with and without weight loss. Active management of the adverse events that may be associated with nintedanib is important to help patients remain on therapy.

## Supplementary information

**Additional file 1: Supplemental Appendix 1.** Statistical analyses.

**Additional file 2: Supplemental Appendix 2.** List of investigators [1**].**

**Additional file 3: Supplemental Table 1.** Baseline characteristics in subgroups of patients by BMI (< 25; ≥25 to < 30; ≥30 kg/m^2^) at baseline.

**Additional file 4: Supplemental Table 2.** Outcomes in subgroups of patients by BMI below and at least the median at baseline.

**Additional file 5: Supplemental Table 3.** Annual rate of decline in FVC (mL/year) over 52 weeks in the overall trial population.

**Additional file 6: Supplemental Table 4.** Baseline characteristics in subgroups of patients by weight loss ≤5 and > 5% over 52 weeks (based on the annual rate of decline in weight).

**Additional file 7: Supplemental Figure 1.** Scatter plots showing the correlation between BMI at baseline and FVC (mL) at baseline (A) and between BMI at baseline and the rate of decline in FVC (mL/year) assessed over 52 weeks (B).

**Additional file 8: Supplemental Figure 2.** Annual rate of decline in FVC (mL/year) over 52 weeks in subgroups of patients by weight loss over 52 weeks (based on the change from baseline in weight at week 52).

## Data Availability

All data relevant to this analysis are included in the article or uploaded as supplementary information.

## Abbreviations

BMI: Body mass index
DLco: Diffusing capacity of the lungs for carbon monoxide
FVC: Forced vital capacity
HRCT: High-resolution computed tomography
IPF: Idiopathic pulmonary fibrosis
MedDRA: Medical Dictionary for Regulatory Activities
SGRQ: St. George’s Respiratory Questionnaire
