# Supplementary material for: Analysis of body mass index, weight loss and progression of idiopathic pulmonary fibrosis
Source: Respir Res. 2020 Nov 25;21:312. doi: 10.1186/s12931-020-01528-4 (PMC7690188; doi:10.1186/s12931-020-01528-4)
Supplement: Supplementary file 1 — Additional file 1: Supplemental Appendix 1. Statistical analyses. [file 12931_2020_1528_MOESM1_ESM.docx]

**Additional file 1: *Supplemental Appendix 1: Statistical analyses***. Correlations between baseline BMI and baseline FVC (mL) and between baseline BMI and the rate of decline in FVC (mL/year) over 52 weeks were assessed using Spearman correlation coefficients. Absolute changes from baseline in FVC (mL or % predicted) and SGRQ total score over 52 weeks were analysed using a mixed model for repeated measures (MMRM) with fixed effects for trial, treatment, visit, baseline value, subgroup, treatment-by-visit and baseline-by-visit and the interaction term treatment-by-subgroup. The patient effect was assumed to be random. In analyses of time to first acute exacerbation, time to absolute decline in FVC ≥10% predicted or death, and time to death, hazard ratios and confidence intervals were obtained using a Cox’s proportional hazards model adjusted for trial, treatment, sex, age, height and subgroup and the interaction term treatment-by-subgroup.
