# Supplementary material for: Analysis of body mass index, weight loss and progression of idiopathic pulmonary fibrosis
Source: Respir Res. 2020 Nov 25;21:312. doi: 10.1186/s12931-020-01528-4 (PMC7690188; doi:10.1186/s12931-020-01528-4)
Supplement: Supplementary file 2 — Additional file 2: Supplemental Appendix 2. List of investigators [1]. [file 12931_2020_1528_MOESM2_ESM.docx]

***Supplemental Appendix 2: List of investigators [1]***

***Australia:*** T. Corte, Royal Prince Alfred Hospital, Camperdown, New South Wales; H. Davies, Repatriation General Hospital, Daw Park, South Australia; I. Glaspole, Alfred Hospital, Melbourne, Victoria; J. Mulder, Frankston Hospital, Frankston, Victoria; E. Veitch, Concord Repatriation General Hospital, Concord, New South Wales. ***Belgium:*** P. De Vuyst, Erasme University Hospital, Brussels; G. Liistro, Cliniques Universitaires Saint-Luc, Brussels; Y. Sibille, Cliniques Universitaires UCL de Mont-Godinne, Yvoir; W. Vincken, Universitair Ziekenhuis Brussel, Brussels; W. Wuyts, Universitair Ziekenhuis Leuven Gasthuisberg Campus, Leuven. ***Canada:*** C. Fell, Peter Lougheed Centre, Calgary, Alberta; P. Hernandez, Queen Elizabeth II Health Sciences Centre, Halifax, Nova Scotia; M. Kolb, St. Joseph’s Healthcare, Hamilton, Ontario. ***Chile:*** A. Undurraga, Instituto Nacional de Torax, Santiago. ***China:*** C. Bai, Zhongshan Hospital Fudan University, Shanghai; P. Chen, The General Hospital of Shenyang Military Command, Shenyang; Z. Gao, Peking University People’s Hospital, Beijing; J. Kang, The First Hospital of Chinese Medical University, Shenyang; H. Li, Shanghai Pulmonary Hospital, Shanghai; Z. Li, Xijing Hospital, 4^th^ Military Medical University, Xi’an; H. Wan, Shanghai Ruijin Hospital, Shanghai; H. Wang, Beijing Friendship Hospital, Beijing; F. Wen, West China Hospital Sichuan University, Chengdu; Q. Xiao, Xiangya Hospital, Central South University, Changsha; Z. Xu, Peking Union Medical College Hospital, Beijing; W. Zhang, The First Affiliated Hospital of Nanchang University, Nanchang; X. Zheng, General Hospital of Ningxia Medical University, Yinchuan; H. Zhu, Shanghai Huadong Hospital, Shanghai. ***Czech Republic:*** N. Pauk, University Hospital Na Bulovce, Praha; P. Reiterer, Masaryk Hospital, Usti nad Labem; M. Vasakova, Thomayer’s Hospital, Clinic of Pneumology, Praha. ***Finland:*** U. Hodgson, HYKS Keuhkosairauksien tutkimusyksikkö, Helsinki. ***France:*** A. Bourdin, Hôpital A. Villeneuve, Pneumologie, Montpellier; J. Cadranel, Hôpital Tenon, Pneumologie, Paris; P. Camus, Hôpital du Bocage, Pneumologie, Dijon; P. Chanez, Hôpital Nord, Pneumologie, Marseille; V. Cottin, Hôpital Louis Pradel, Pneumologie, Lyon; B. Crestani, Hôpital Bichat, Pneumologie, Paris; D. Israel-Biet, Hôpital Européen Georges-Pompidou, Pneumologie, Paris; S. Jouneau, Hôpital Pontchaillou, Pneumologie, Rennes; F. Lebargy, Hôpital Maison Blanche, Pneumologie, Reims; C. H. Marquette, Hôpital Pasteur, Pneumologie, Nice; G. Prévot, Hôpital Larrey, Pneumologie, Toulouse; D. Valeyre, Hôpital Avicenne, Pneumologie, Bobigny; B. Wallaert, Hôpital Calmette, Pneumologie, Lille. ***Germany:*** R. Bonnet, Zentralklinik Bad Berka GmbH, Bad Berka; U. Costabel, Ruhrlandklinik, Essen; S. Gläser, Ernst-Moritz-Arndt-Universtität, Greifswald; C. Grohé, Evangelische Lungenklinik Berlin; A. Guenther, Universitätsklinikum Gießen, Gießen; P. Hammerl, Fachklinik für Lungenerkrankugen, Immenhausen; G. Höffken, Uniklinik Carl Gustav Carus, Dresden; C. Karagiannidis, Kliniken der Stadt Koeln, Koeln; J. Kirschner, CIMS Studienzentrum Bamberg UG, Bamberg; A. Kirsten, LungenClinic, Grosshansdorf; S. Korn, Johannes-Gutenberg-Universtitätsklinik, Mainz; M. Kreuter, Universitätsklinikum Heidelberg, Heidelberg; J. Müller-Quernheim, Klinik der Albert-Ludwig-Universität, Freiburg; C. Neurohr, Klinikum Großhadern der L.M.-Universtität, München; M. Pfeifer, Krankenhaus, Donaustauf; N. Schönfeld, HELIOS-Kliniken Emil von Behring, Berlin; R. Wiewrodt, Universitätsklinikum Münster, Medizinischen Poliklinik D, Münster. ***Greece:*** K. Antoniou, University Hospital of Heraklion, University Pulmonology Cl, Heraklion, Crete; Z. Daniil, University Hospital of Larissa, Pulmonology Clinic, Larissa; F. Diamantea, General Hospital of Athens “Sismanoglio”, 3^rd^ Pneumonology Department, Athens; N. Koulouris, Athens Hospital of Chest Diseases “Sotiria”, Athens; G. Mathioudakis, General Hospital of Nikaia, Pulmonology Clinic, Nikaia. ***India:*** A. G. Ghosal, National Allergy Asthma Bronchitis Institute, Kolkata; S. Kadappa Shivappa, Chest and Maternity Centre, Bangalore; M. M. Kawedia, Jehangir Hospital, Pune; P. Khatavkar, King Edward Memorial Hospital, Mumbai; A. Kumar, Asthma Bhawan, Jaipur; P. Mehta, Mehta Hospital and Cardiopulmonary Care Centre, Ahmedabad; V. Singh, SMS Medical College and Hospital, Jaipur; K. Srikanth, PSG Hospitals, Coimbatore; H. Thakker, Qualysure Clinical Research Solution, Ahmedabad; Z. Udwadia, P.D. Hinduja National Hospital and Medical Research Center, Mumbai. ***Ireland:*** J. Egan, Mater Misericordiae University Hospital, Department of Respiratory Medicine, Dublin. ***Israel:*** G. Fink, Kaplan Medical Center, Pulmonary Institute, Rehovot; M. R. Kramer, Rabin Medical Center, Petah Tiqwa; M. Yigla, Rambam Medical Center, Haifa. ***Italy:*** C. Agostini, Università degli Studi, Dipartimento di Medicina Clinica e Sperimentale, Padova; F. De Benedetto, P.O. Clinicizzato SS. Annunziata, U.O. di Pneumologia, Chieti; S. Harari, Ospedale S. Giuseppe Fatebenefratelli, Pneumologia, Milano; F. Luppi, Università Modena e Reggio Emilia, Malattie dell’Apparato Respiratorio, Modena; P. L. Paggiaro and L. M. Tavanti, Azienda Ospedaliera Universitaria Pisana, Ospedale Cisanello, Fisiop. Respiratoria, Pisa; A. Pesci, Nuovo Ospedale “S. Gerardo”, U.O. Pneumologia, Monza; V. Poletti, Ospedale Morgagni-Pierantoni, Dipartimento Toracico U.O. Pneumologia, Forli; P. Rottoli, Presidio Ospedale Le Scotte, Clinica Pneumologica, Siena; C. Saltini, Policlinico Università Tor Vergata, Malattie Respiratorie, Roma; A. Sanduzzi Zamparelli, Università Federico II – II Clinica Pneumologica, Napoli; C. Vancheri, A.O.U. Policlinico Dipartimento di Medicina Interna e Special. Sez. Mal. Resp., Catania. ***Japan:*** M. Bando, Jichi Medical University Hospital, Pulmonary Medicine, Tochigi; Y. Hasegawa, Nagoya University Hospital, Respiratory Medicine, Aichi; K. Hashimoto, Tottori University Hospital, Respiratory Medicine, Tottori; S. Homma, Toho University Omori Medical Center, Respiratory Medicine, Tokyo; N. Inase, Tokyo Medical and Dental University, Pulmonary Medicine, Tokyo; Y. Inoue and T. Arai, National Hospital Organization Kinki-Chuo Chest Medical Center, Department of Internal Medicine, Osaka; S. Izumi, National Center for Global Health and Medicine, Respiratory Medicine, Tokyo; T. Kawamura, Himeji Medical Center, Respiratory Tract Medicine, Hyogo; K. Kishi, Toranomon Hospital, Department of Respiratory Medicine, Respiratory Center, Tokyo; Y. Kondo, Tosei General Hospital, Department of Allergy and Respiratory Medicine, Aichi; K. Kuwano, The Jikei University Hospital, Division of Respiratory Disease, Department of Internal Medicine, Tokyo; Y. Miura, Nippon Medical School Hospital, Respiratory Medicine, Tokyo; Y. Nishioka, Tokuschima University Hospital, Respiratory Medicine and Rheumatology, Tokushima; O. Nishiyama, Kinki University Hospital, Respiratory Medicine and Allergy, Osaka; T. Ogura, Kanagawa Cardiovascular and Respiratory Center, Respiratory Medicine, Kanagawa; S. Ohkouchi, Tohoku University Hospital, Department of Respiratory Medicine, Miyagi; T. Saito, Ibarakihigashi National Hospital, Ibaraki Department of Respiratory Medicine, Naka; Y. Setoguchi, Tokyo Medical University Hospital, Department of Respiratory Medicine, Respiratory Center, Tokyo; J. Shindoh, Ogaki Municipal Hospital, Respiratory Med, Gifu; Y. Taguchi, Tenri Hospital, Department of Respiratory Medicine, Nara; M. Tanakadate, Toranomon Hospital Kajigaya, Pulmonary and Critical Care Medicine, Kanagawa; K. Tomii, Kobe City Medical Center General Hospital, Respiratory Medicine, Hyogo; Y. Sugita, Saitama Cardiovascular and Respiratory Center, Respiratory Medicine, Saitama; T. Yamaguchi, JR Tokyo General Hospital, Respiratory Medicine, Tokyo; K. Yoshimori, Fukujuji Hospital, Respiratory Medicine, Tokyo. ***Korea:*** S. H. Jeong, Gachon University Gil Hospital, Incheon; D. S. Kim, Asan Medical Center, Seoul; Y. W. Kim, Seoul National University Hospital, Seoul; C. S. Park, Soon Chun Hyang University Hospital Bucheon, Bucheon; J. S. Song, The Catholic University of Korea, Yeouido St. Mary’s Hospital, Seoul; S. T. Uh, Soon Chun Hyang University Hospital Seoul, Seoul. ***Mexico:*** M. Selman, Instituto Nacional de Enfermedades Respiratorias, Ciudad de Mexico. ***Netherlands:*** P. Bresser, Onze Lieve Vrouwe Gasthius, Amsterdam; J. C. Grutters, St. Antonius Ziekenhuis, Nieuwegein, Utrecht; M. S. Wijsenbeek, Erasmus Medisch Centrum, Rotterdam. ***Portugal:*** A. Arrobas, Centro Hospitalar e Universitário de Coimbra, EPE, Centro Hospitalar Coimbra, Pneumology Department, Coimbra; J. Cardoso, Centro Hospitalar Lisboa Central, EPE, Hospital de Santa Marta, Pneumology Department, Lisboa; R. Costa, Hospital Fernando Fonseca, EPE, Pneumology Department, Amadora; A. Morais, Centro Hospitalar São João, EPE, Pneumology Department, Porto; S. Neves, Centro Hospitalar de Vila Nova Gaia/Espinho, Pneumology Department, Vila Nova de Gaia; M. Serrado, Centro Hospitalar Lisboa Norte, EPE, Hospital Pulido Valente, Pneumology II Department, Lisboa. ***Russia:***  M. Ilkovick, Scientific Research Institute of Pulmonology, Saint Petersburg; A. Vizel, GOU VPO Kazan State Medical University of Roszdrav, Kazan. ***Spain:*** I. Alfageme Michavila, Hospital de Valme, Sevilla; J. Ancochea, Hospital Universitario de la Princesa, Madrid; D. Castillo Villegas, Hospital de la Santa Cruz y San Pablo, Barcelona; M. Molina-Molina, Ciutat Sanitaria Universitaria Bellvitge, Barcelona; F. Morell, Hospital Vall d’Hebron, Barcelona; A. Xaubet, Hospital Clinic i Provincial de Barcelona, Neumología, Barcelona. ***Turkey:*** S. Aktogu Ozkan, Dr. Suat Seren Gogus Hastaliklari ve Gogus Cerrahisi Egit ve Arastirma Hastanesi, Konak/İzmir; O. Kayacan, Ankara Universitesi Tip Fakültesi Gögüs Hastaliklari, Ankara; G. Ongen, Istanbul Universitesi Cerrahpasa Tip Fakultesi Gogus Hastaliklari, Istanbul; N. Mogulkoc, Ege Universitesi Tip Fakultesi Gögüs Hastaliklari ABD, İzmir; E. Tuncay, Yedikule Gogus Hastaliklari ve Gogus Cer Egitim ve Arastirma Hastanesi, Istanbul. ***United Kingdom:*** P. Beirne, St James’s University Hospital, Respiratory Medicine, Leeds; H. Bettinson, Churchill Hospital, Oxford Centre for Respiratory Medicine, Oxford; P. Burge, Birmingham Heartlands Hospital, Department of Respiratory Medicine, Birmingham; O. Dempsey, Aberdeen Royal Infirmary, Respiratory Department, Aberdeen; T. Maher, Royal Brompton Hospital, Interstitial Lung Disease Unit, London; A. Millar, Southmead Hospital, North Bristol Lung Centre, Bristol; L. Spencer, University Hospital Aintree, Aintree Chest Centre, Liverpool; D. Thickett, Queen Elizabeth Hospital, Lung Investigation Unit, Birmingham. ***United States:*** J. Alvarez, Loess Hill Research Center, Council Bluffs, Iowa; C. Andrews, Diagnostics Research Group, San Antonio, Texas; O. Bajwa, Allegheny General Hospital, Allegheny-Singer Research Institute, Pittsburgh, Pennsylvania; A. Baker, Lynchburg Pulmonary Associates, Lynchburg, Tennessee; R. Baughman, University of Cincinnati Medical Center, Cincinnati, Ohio; J. Belperio, David Geffen School of Medicine at UCLA, Los Angeles, California; J. Bradley, Consultants in Pulmonary Medicine, Olathe, Kansas; H. Collard, University of California at San Francisco, San Francisco, California; F. Cordova, Temple Lung Center Ambulatory Care Center, Philadelphia, Pennsylvania; C. Daniels, Mayo Clinic College of Medicine, Rochester, New York; J. de Andrade, University of Alabama, Division of Pulmonary, Birmingham, Alabama; K. Dushay, Rhode Island Hospital, Providence, Rhode Island; R. Enelow, Dartmouth Hitchock Medical Center, Lebanon, Pennsylvania; N. Ettinger, Cardiopulmonary Associates, Chesterfield, Missouri; K. Gibson, University of Pittsburgh School of Medicine, Pittsburgh, Pennsylvania; M. Gotfried, Pulmonary Associates Clinical Trials, Phoenix, Arizona; A. Hajari Case, Georgia Clinical Research, Austell, Georgia; D. Hotchkin, Oregon Clinic, PC, Portland, Oregon; J. T. Huggins, Medical University of South Carolina, Charleston, South Carolina; M. Kaye, Minnesota Lung Center, Ltd., Minneapolis, Minnesota; C. Kershaw, University of Texas Southwestern Medical Center, Dallas, Texas; S. Kureishy, Metroplex Pulmonary and Sleep Centre, McKinney, Texas; L. Lancaster, Vanderbilt University Medical Center, Nashville, Tennessee; D. Lederer, New York Presbyterian Hospital, New York, New York; Y. Mageto, Vermont Lung Center, Colchester, Vermont; J. Masson, Pulmonary and Critical Care Associates, Albany, New York; K. Meyer, University of Wisconsin Hospital and Clinics, Madison, Wisconsin; P. Mohabir, Stanford Medical Center Pulmonary and Critical Care Clinic, Stanford, California; L. Morrison, Duke University Medical Center, Durham, North Carolina; S. Nathan, Inova Fairfax Hospital, Falls Church, Virginia; I. Noth, University of Chicago, Chicago, Illinois; D. Oelberg, Western Connecticut Medical Group P.C., Danbury, Connecticut; F. Rahaghi, Cleveland Clinic Florida, Weston, Florida; D. Riley, University of Medicine and Dentistry of NJ, New Brunswick, New Jersey; A. Rizzo, Lung Health and Sleep Enhancement Center, LLC, Newark, New Jersey; M. Rossman, Hospital of the University of Pennsylvania, Philadelphia, Pennsylvania; J. Ruzi, Arizona Pulmonary Specialists, Scottsdale, Arizona; P. Sachs, Pulmonary Associates of Stamford, Stamford, Connecticut; T. Schaumberg, Oregon Clinic, PC, Portland, Oregon; M. Scholand, Lung Health Research Center, Salt Lake City, Utah; C. Schroeder, Sansum Clinic, Santa Barbara, California; F. Seifer, FDC Seifer PLC Pulmonary, Shelbyville, Illinois; J. Shea, DCOL Center for Clinical Research, Longview, Texas; D. Sinkowitz, UCLA David Geffen School of Medicine, Torrance, California; J. Tabak, Miami Research Associates, South Miami, Florida; J. Taylor, MultiCare Pulmonary Specialists, Tacoma, Washington; J. Thompson, Baptist Pulmonary and Critical Care Associates, Lexington, Kentucky; C. Thurm, Jamaica Hospital Medical Center, Department of Medicine, Jamaica, New York; J. Tita, ID Clinical Research, Ltd., Toledo, Ohio; M. Wencel, Via Christi Clinic, PA, Wichita, Kansas; J. Westerman, Jasper Summit Research, LLC, Jasper, Alabama.

**Reference**

1. Richeldi L, du Bois RM, Raghu G, et al. Efficacy and safety of nintedanib in idiopathic pulmonary fibrosis. N Engl J Med 2014;370(22):2071–82.
