# Supplementary material for: Analysis of body mass index, weight loss and progression of idiopathic pulmonary fibrosis
Source: Respir Res. 2020 Nov 25;21:312. doi: 10.1186/s12931-020-01528-4 (PMC7690188; doi:10.1186/s12931-020-01528-4)
Supplement: Supplementary file 3 — Additional file 3: Supplemental Table 1. Baseline characteristics in subgroups of patients by BMI (< 25; ≥25 to < 30; ≥30 kg/m2) at baseline. [file 12931_2020_1528_MOESM3_ESM.docx]

**Supplemental Table 1.** Baseline characteristics in subgroups of patients by BMI (<25; ≥25 to <30; ≥30 kg/m^2^) at baseline.

|  | **BMI <25 kg/m^2^ at baseline** | | **BMI ≥25 and <30 kg/m^2^ at baseline** | | **BMI ≥30 kg/m^2^ at baseline** | |
| --- | --- | --- | --- | --- | --- | --- |
|  | **Nintedanib (n=167)** | **Placebo (n=140)** | **Nintedanib (n=285)** | **Placebo (n=168)** | **Nintedanib (n=186)** | **Placebo (n=115)** |
| Age, years, mean (SD) | 68.2 (8.5) | 67.0 (8.4) | 66.6 (8.2) | 67.3 (7.7) | 65.2 (7.5) | 66.4 (7.6) |
| Male, n (%) | 122 (73.1) | 107 (76.4) | 243 (85.3) | 132 (78.6) | 142 (76.3) | 95 (82.6) |
| Weight, kg, mean (SD) | 62.0 (8.5) | 64.1 (9.0) | 78.2 (9.0) | 78.4 (9.5) | 96.3 (13.9) | 96.7 (14.0) |
| Body mass index, kg/m^2^, mean (SD) | 22.9 (1.7) | 23.0 (1.8) | 27.5 (1.4) | 27.4 (1.4) | 33.6 (3.3) | 33.5 (3.2) |
| Race, n (%) |  |  |  |  |  |  |
| White | 44 (26.3) | 54 (38.6) | 164 (57.5) | 101 (60.1) | 152 (81.7) | 93 (80.9) |
| Asian | 105 (62.9) | 80 (57.1) | 73 (25.6) | 42 (25.0) | 16 (8.6) | 6 (5.2) |
| Black | 0 | 0 | 1 (0.4) | 0 (0.0) | 1 (0.5) | 0 (0.0) |
| Missing* | 18 (10.8) | 6 (4.3) | 47 (16.5) | 25 (14.9) | 17 (9.1) | 16 (13.9) |
| Time since diagnosis of IPF, years, mean (SD) | 1.63 (1.43) | 1.46 (1.28) | 1.63 (1.34) | 1.59 (1.35) | 1.68 (1.33) | 1.68 (1.28) |
| Current or former smoker, n (%) | 108 (64.7) | 88 (62.9) | 219 (76.8) | 122 (72.6) | 137 (73.7) | 91 (79.1) |
| FVC, mL, mean (SD) | 2535 (684) | 2603 (751) | 2866 (767) | 2782 (818) | 2640 (762) | 2800 (857) |
| FVC, % predicted, mean (SD) | 81.2 (18.3) | 78.3 (17.5) | 81.9 (17.3) | 80.5 (18.7) | 75.0 (16.5) | 78.7 (18.6) |
| SpO_2_, %, mean (SD) | 96.2 (2.0) | 96.2 (2.0) | 96.0 (2.3) | 95.8 (1.9) | 95.3 (2.5) | 95.3 (2.2) |
| DLco^†^, % predicted, mean (SD) | 44.8 (13.4) | 44.9 (14.6) | 48.7 (12.7) | 46.7 (12.4) | 47.9 (14.5) | 49.9 (12.8) |
| SGRQ total score, mean (SD) | 38.1 (20.1) | 38.8 (19.1) | 36.6 (18.2) | 37.4 (18.5) | 45.3 (18.5) | 43.8 (17.4) |
| Emphysema^‡^, n (%) | 79 (47.3) | 57 (40.7) | 114 (40.0) | 65 (38.7) | 61 (32.8) | 44 (38.3) |

*In France, regulation did not permit the collection of data on race.

^†^Corrected for haemoglobin.

^‡^Based on qualitative assessment of HRCT scans by the investigator. Emphysema was classified as present or absent.
