# Supplementary material for: Analysis of body mass index, weight loss and progression of idiopathic pulmonary fibrosis
Source: Respir Res. 2020 Nov 25;21:312. doi: 10.1186/s12931-020-01528-4 (PMC7690188; doi:10.1186/s12931-020-01528-4)
Supplement: Supplementary file 4 — Additional file 4: Supplemental Table 2. Outcomes in subgroups of patients by BMI below and at least the median at baseline. [file 12931_2020_1528_MOESM4_ESM.docx]

**Supplemental Table 2.** Outcomes in subgroups of patients by BMI below and at least the median at baseline.

|  | **BMI <27 kg/m^2^ at baseline** | | **BMI ≥27 kg/m^2^ at baseline** | |
| --- | --- | --- | --- | --- |
|  | **Nintedanib (n=278)** | **Placebo (n=208)** | **Nintedanib (n=360)** | **Placebo (n=215)** |
| Annual rate of decline in FVC (mL/year) over 52 weeks | -108.5 (16.2) | -266.2 (18.7) | -117.7 (14.8) | -183.1 (19.1) |
| Difference versus placebo (95% CI) | 157.8 (109.1, 206.4) | | 65.4 (17.8, 112.9) | |
| p-value for treatment-by-time-by-subgroup interaction | 0.0074 | | | |
| Absolute change from baseline in FVC (mL) over 52 weeks | -114.8 (18.4) | -264.3 (21.2) | -101.2 (15.4) | -167.3 (19.8) |
| Difference versus placebo (95% CI) | 149.5 (94.4, 204.5) | | 66.0 (16.8, 115.2) | |
| p-value for treatment-by-subgroup interaction | 0.64 | | | |
| Absolute change from baseline in FVC (% predicted) over 52 weeks | -3.7 (0.6) | -8.0 (0.6) | -3.0 (0.4) | -4.8 (0.6) |
| Difference versus placebo (95% CI) | 4.3 (2.6, 6.0) | | 1.8 (0.4, 3.2) | |
| p-value for treatment-by-subgroup interaction | 0.37 | | | |
| Absolute change from baseline in SGRQ total score over 52 weeks | 4.1 (1.0) | 6.7 (1.2) | 3.5 (0.9) | 3.9 (1.1) |
| Difference versus placebo (95% CI) | -2.6 (-5.7, 0.6) | | -0.4 (-3.2, 2.3) | |
| p-value for treatment-by-subgroup interaction | 0.80 | | | |
| Patients with absolute decline in FVC ≥10% predicted or death at week 52, n (%) | 74 (26.6) | 94 (45.2) | 99 (27.5) | 81 (37.7) |
| HR (95% CI) | 0.49 (0.36, 0.67) | | 0.71 (0.53, 0.95) | |
| p-value for treatment-by-subgroup interaction | 0.14 | | | |
| Patients with ≥1 acute exacerbation of IPF over 52 weeks | 17 (6.1) | 19 (9.1) | 14 (3.9) | 13 (6.0) |
| HR (95% CI) | 0.65 (0.34, 1.26) | | 0.65 (0.31, 1.40) | |
| p-value for treatment-by-subgroup interaction | 0.96 | | | |
| Deaths over 52 weeks, n (%) | 14 (5.0) | 21 (10.1) | 21 (5.8) | 12 (5.6) |
| HR (95% CI) | 0.46 (0.24, 0.92) | | 1.07 (0.53, 2.19) | |
| p-value for treatment-by-subgroup interaction | 0.11 | | | |

Changes from baseline are adjusted means (SE). HR, hazard ratio.
