# Supplementary material for: Analysis of body mass index, weight loss and progression of idiopathic pulmonary fibrosis
Source: Respir Res. 2020 Nov 25;21:312. doi: 10.1186/s12931-020-01528-4 (PMC7690188; doi:10.1186/s12931-020-01528-4)
Supplement: Supplementary file 5 — Additional file 5: Supplemental Table 3. Annual rate of decline in FVC (mL/year) over 52 weeks in the overall trial population. [file 12931_2020_1528_MOESM5_ESM.docx]

**Supplemental Table 3.** Annual rate of decline in FVC (mL/year) over 52 weeks in the overall trial population.

|  | **Original analysis*^1^** | | **Analysis adjusted for BMI at baseline^†^** | | **Analysis adjusted for weight loss over 52 weeks^‡^** | |
| --- | --- | --- | --- | --- | --- | --- |
|  | **Nintedanib (n=638)** | **Placebo (n=423)** | **Nintedanib (n=638)** | **Placebo (n=423)** | **Nintedanib (n=638)** | **Placebo (n=423)** |
| Adjusted mean (SE) rate of decline in FVC (mL/year) over 52 weeks | -113.6 (11.0) | -223.5 (13.4) | -112.9 (11.8) | -228.1 (14.2) | -113.0 (11.8) | -227.8 (14.2) |
| Difference versus placebo (95% CI) | 109.9 (75.9, 144.0) | | 115.2 (79.0, 151.4) | | 114.8 (78.6, 151.0) | |
| p-value | <0.001 | | <0.001 | | <0.001 | |

*Random coefficient regression with fixed effects for trial, treatment, sex, age, height and random effect of patient-specific intercept and time. **^†^**Random coefficient regression with fixed effects for trial, treatment, sex, age, height, race and BMI at baseline (<25, ≥25 to <30, ≥30 kg/m^2^) and random effect of patient-specific intercept and time. ^‡^Random coefficient regression with fixed effects for trial, treatment, sex, age, height, race and weight loss over 52 weeks (≤5%, <5%) and random effect of patient-specific intercept and time.
