# Supplementary material for: Analysis of body mass index, weight loss and progression of idiopathic pulmonary fibrosis
Source: Respir Res. 2020 Nov 25;21:312. doi: 10.1186/s12931-020-01528-4 (PMC7690188; doi:10.1186/s12931-020-01528-4)
Supplement: Supplementary file 6 — Additional file 6: Supplemental Table 4. Baseline characteristics in subgroups of patients by weight loss ≤5 and > 5% over 52 weeks (based on the annual rate of decline in weight). [file 12931_2020_1528_MOESM6_ESM.docx]

**Supplemental Table 4.** Baseline characteristics in subgroups of patients by weight loss ≤5% and >5% over 52 weeks (based on the annual rate of decline in weight).

|  | **Weight loss ≤5%** | | **Weight loss >5%** | |
| --- | --- | --- | --- | --- |
|  | **Nintedanib (n=397)** | **Placebo (n=338)** | **Nintedanib (n=241)** | **Placebo (n=85)** |
| Age, years, mean (SD) | 65.7 (7.9) | 66.4 (8.1) | 68.1 (8.2) | 69.1 (6.6) |
| Male, n (%) | 330 (83.1) | 277 (82.0) | 177 (73.4) | 57 (67.1) |
| Weight, kg, mean (SD) | 79.6 (16.5) | 79.4 (16.4) | 78.6 (16.7) | 75.6 (17.0) |
| Body mass index, kg/m^2^, mean (SD) | 27.9 (4.5) | 27.8 (4.5) | 28.4 (4.7) | 27.1 (5.0) |
| Race, n (%) |  |  |  |  |
| White | 223 (56.2) | 196 (58.0) | 137 (56.8) | 52 (61.2) |
| Asian | 126 (31.7) | 104 (30.8) | 68 (28.2) | 24 (28.2) |
| Black | 1 (0.3) | 0 | 1 (0.4) | 0 |
| Missing* | 47 (11.8) | 38 (11.2) | 35 (14.5) | 9 (10.6) |
| Time since diagnosis of IPF, years, mean (SD) | 1.6 (1.4) | 1.6 (1.3) | 1.8 (1.4) | 1.5 (1.2) |
| Current or former smoker, n (%) | 287 (72.3) | 248 (73.4) | 177 (73.4) | 53 (62.4) |
| FVC, mL, mean (SD) | 2832 (736) | 2804 (815) | 2518 (753) | 2424 (721) |
| FVC, % predicted, mean (SD) | 81.0 (17.8) | 80.1 (18.1) | 77.6 (17.0) | 76.0 (18.4) |
| SpO_2_, %, mean (SD) | 96.0 (2.4) | 95.8 (2.1) | 95.6 (2.2) | 95.8 (1.8) |
| DLco^†^, % predicted, mean (SD) | 48.7 (13.6) | 47.6 (12.7) | 45.3 (13.1) | 44.3 (15.7) |
| SGRQ total score, mean (SD) | 37.0 (19.2) | 38.0 (18.5) | 43.7 (18.4) | 46.0 (17.5) |
| Emphysema^‡^, n (%) | 172 (43.3) | 142 (42.0) | 82 (34.0) | 24 (28.2) |

*In France, regulation did not permit the collection of data on race in the INPULSIS trials.

^†^Corrected for haemoglobin.

^‡^Based on qualitative assessment of HRCT scans by the investigator. Emphysema was classified as present or absent.
