# Supplementary material for: Analysis of body mass index, weight loss and progression of idiopathic pulmonary fibrosis
Source: Respir Res. 2020 Nov 25;21:312. doi: 10.1186/s12931-020-01528-4 (PMC7690188; doi:10.1186/s12931-020-01528-4)

**Supplemental Figure 1.** Scatter plots showing the correlation between BMI at baseline and FVC (mL) at baseline (A) and between BMI at baseline and the rate of decline in FVC (mL/year) assessed over 52 weeks (B).

**A**


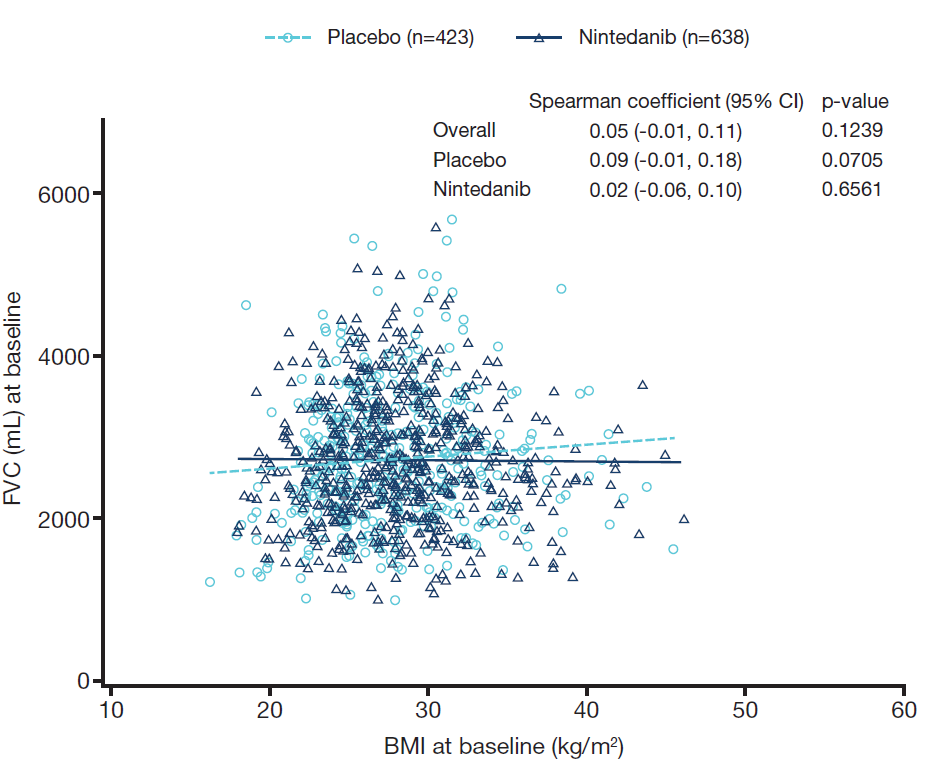


**B**


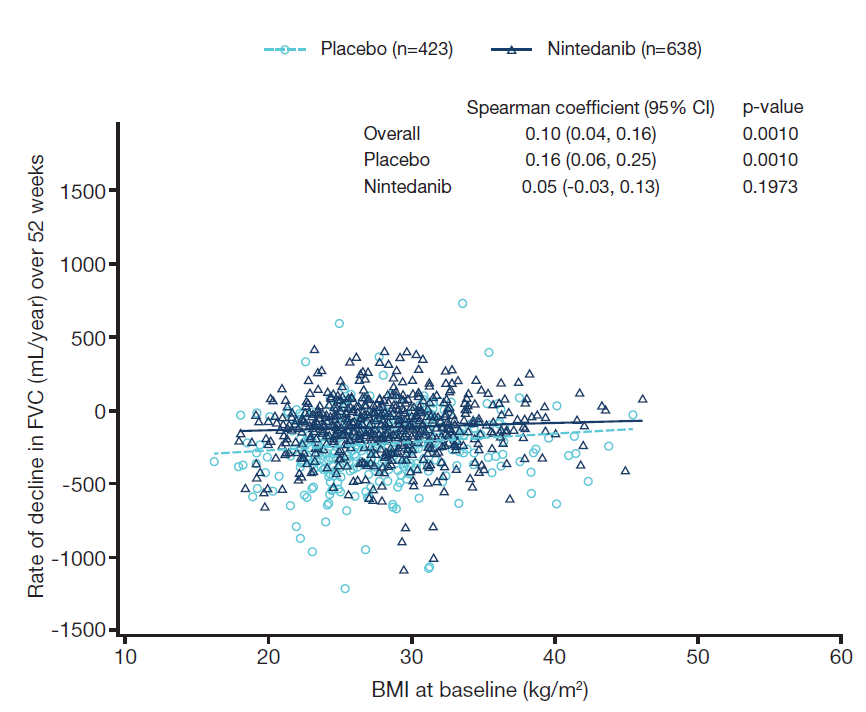

Supplement: Supplementary file 7 — Additional file 7: Supplemental Figure 1. Scatter plots showing the correlation between BMI at baseline and FVC (mL) at baseline (A) and between BMI at baseline and the rate of decline in FVC (mL/year) assessed over 52 weeks (B). [file 12931_2020_1528_MOESM7_ESM.docx]
