# Supplementary material for: Analysis of body mass index, weight loss and progression of idiopathic pulmonary fibrosis
Source: Respir Res. 2020 Nov 25;21:312. doi: 10.1186/s12931-020-01528-4 (PMC7690188; doi:10.1186/s12931-020-01528-4)

**Supplemental Figure 2.** Annual rate of decline in FVC (mL/year) over 52 weeks in subgroups of patients by weight loss over 52 weeks (based on the change from baseline in weight at week 52).


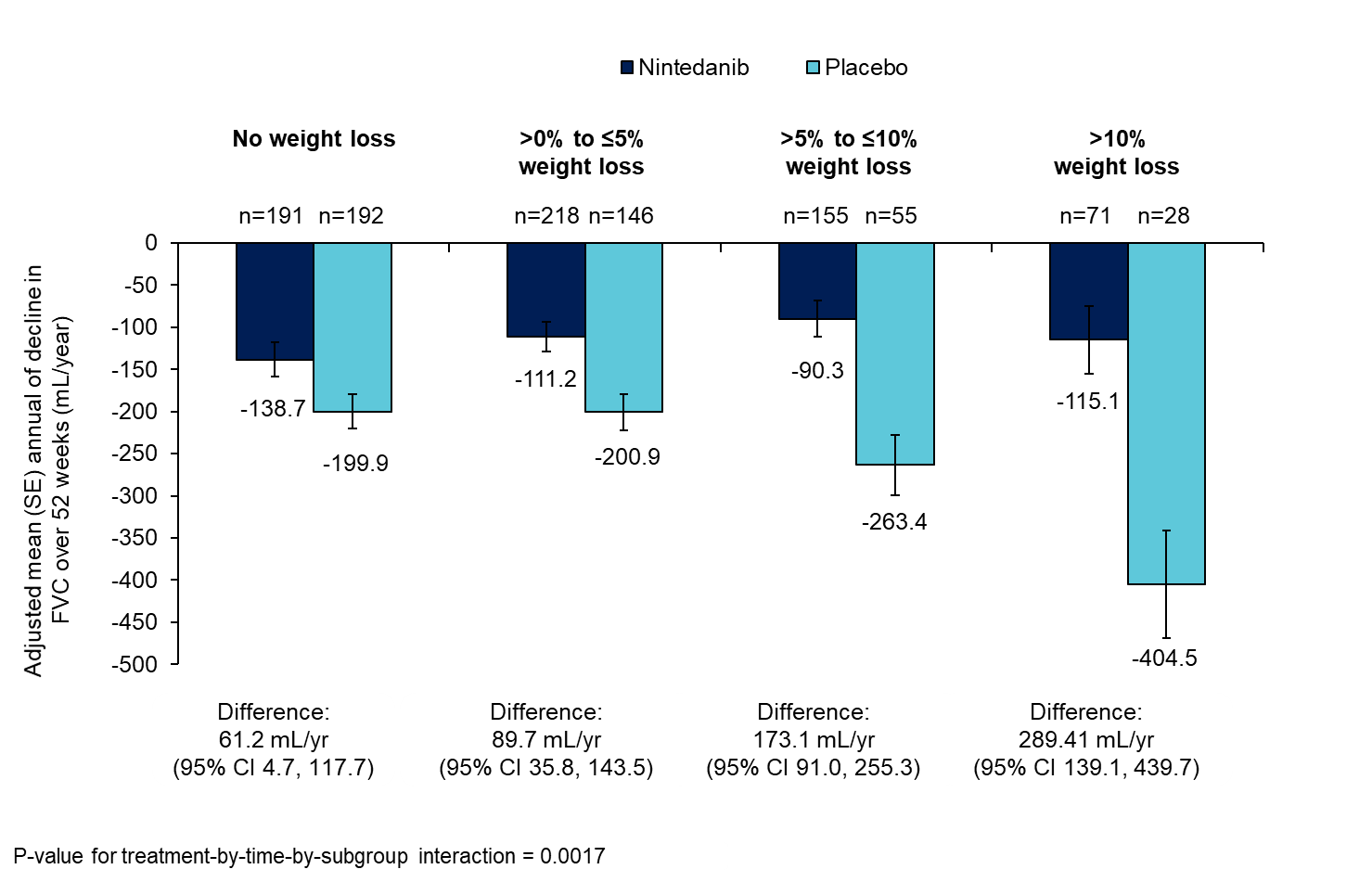

Supplement: Supplementary file 8 — Additional file 8: Supplemental Figure 2. Annual rate of decline in FVC (mL/year) over 52 weeks in subgroups of patients by weight loss over 52 weeks (based on the change from baseline in weight at week 52). [file 12931_2020_1528_MOESM8_ESM.docx]
